# The molecular mechanism of ferroptosis and its role in COPD

**DOI:** 10.3389/fmed.2022.1052540

**Published:** 2023-01-06

**Authors:** Dandan Meng, Chengfeng Zhu, Ruixue Jia, Zongxin Li, Wantao Wang, Suhua Song

**Affiliations:** ^1^Department of Traditional Chinese Medicine, Shandong University of Traditional Chinese Medicine, Jinan, China; ^2^Department of Second Department of Haematology, Jinan Haematology Hospital, Jinan, China; ^3^Department of Basic Medicine, Shanghai University of Traditional Chinese Medicine, Shanghai, China

**Keywords:** ferroptosis, COPD, inflammation, iron, lipid peroxidation

## Abstract

Ferroptosis, a new type of cell death, is mainly characterized by intracellular iron accumulation and lipid peroxidation. The complex regulatory network of iron metabolism, lipid metabolism, amino acid metabolism, p53-related signaling, and Nrf2-related signaling factors is involved in the entire process of ferroptosis. It has been reported that ferroptosis is involved in the pathogenesis of neurological diseases, cancer, and ischemia–reperfusion injury. Recent studies found that ferroptosis is closely related to the pathogenesis of COPD, which, to some extent, indicates that ferroptosis is a potential therapeutic target for COPD. This article mainly discusses the related mechanisms of ferroptosis, including metabolic regulation and signaling pathway regulation, with special attention to its role in the pathogenesis of COPD, aiming to provide safe and effective therapeutic targets for chronic airway inflammatory diseases.

In 2012, Dixon (1) first proposed a new type of iron-dependent programmed cell death named ferroptosis. As a new type of cell death, ferroptosis is mainly characterized by intracellular iron accumulation and lipid peroxidation. Ferroptosis differs from other forms of cell death such as apoptosis and necrosis in terms of morphology, biochemical characteristics, and genetics (2, 3). The morphological aspects of ferroptosis are mainly characterized by mitochondrial contraction and increased density of mitochondrial membranes with a decrease or disappearance of mitochondrial cristae and disintegration of the outer membrane (1). The biochemical features of ferroptosis are as follows: accumulation of ROS and iron ions, decreased cysteine uptake and GSH synthesis, activation of the mitogen-activated protein kinase system, and release of arachidonic acid (4). Iron metabolism, lipid metabolism, amino acid metabolism, p53-related signaling factors, and Nrf2-related signaling factors are involved in the entire process of regulating ferroptosis (5). Ferroptosis, a new programmed cell death mode, has been confirmed to be closely related to tumors, central nervous system diseases, arteriosclerosis, acute kidney injury, diabetes, and ischemia–reperfusion injury (6). Recent studies found that ferroptosis is also associated with the onset of chronic obstructive pulmonary disease (COPD) and has the potential to become a new therapeutic target. COPD is a common condition that can be prevented and treated, and it is characterized by persistent respiratory symptoms and airflow restriction; in addition, COPD is caused by airway and alveolar abnormalities due to heavy exposure to harmful particles or gases, and it is affected by host factors such as abnormal pulmonary development (7). COPD has developed into a major chronic respiratory disease that seriously threatens human health. Related studies showed that the disruption of iron homeostasis is related to the pathogenesis of COPD (8). With further study, the role of ferroptosis in the pathogenesis of COPD has gradually been revealed, and appropriate intervention in ferroptosis can delay the COPD process.

## 1. Mechanism of ferroptosis

**Figure 1:**
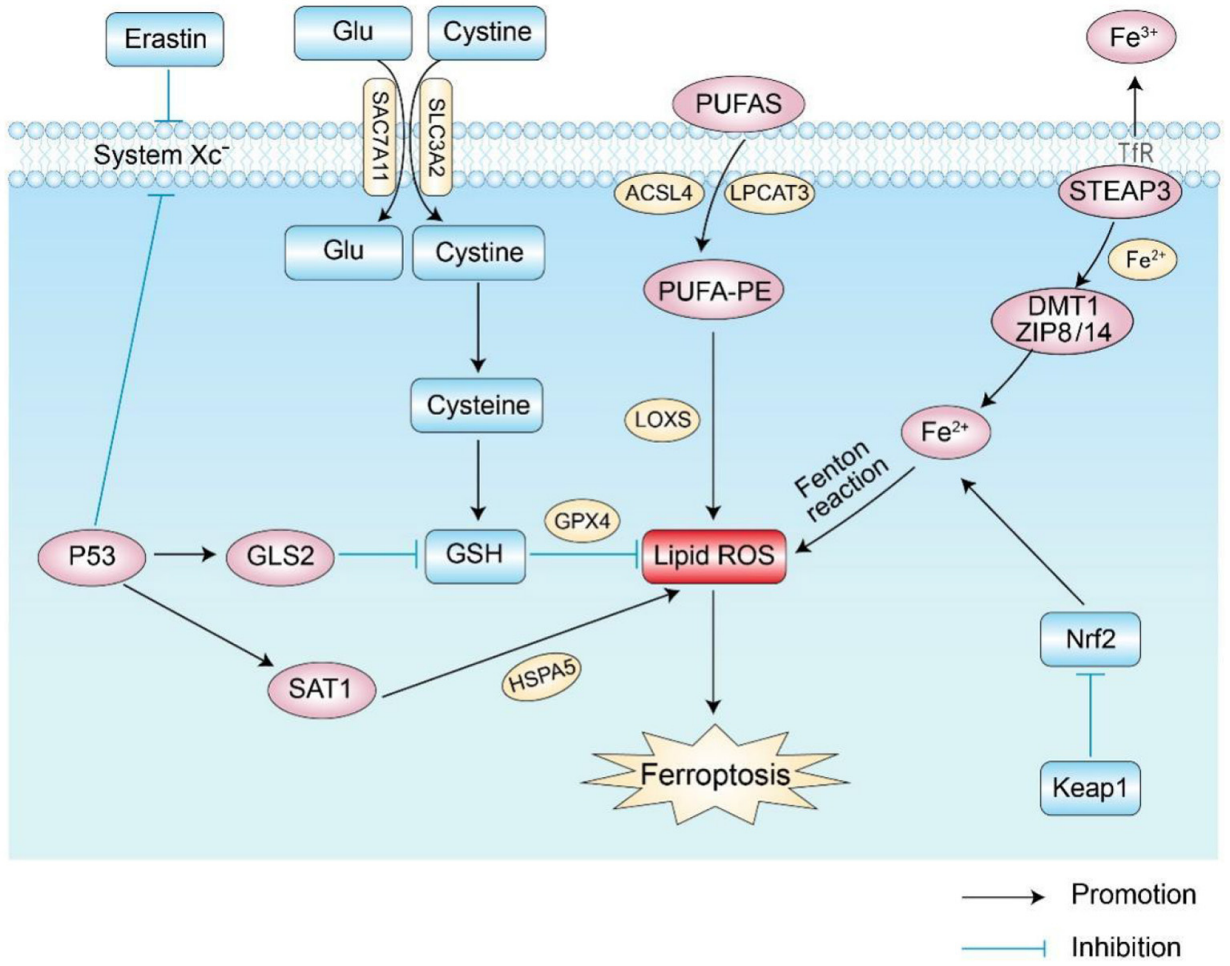
Main regulatory pathways of ferroptosis. There are two main ways of regulating ferroptosis shown in the figure: the first is the pathway of abnormal iron, amino acid, and lipid metabolism; the second involves the related signaling pathways that regulate ferroptosis, such as the P53 and Nrf2 pathways. Glu, glutamic acid; GSH, glutathione; GPX4, glutathione peroxidase 4; Nrf2, Nuclear factor erythroid 2-related factor 2; GLS2, glutaminase 2; LOXs, lipoxygenases; ROS, reactive oxygen species; DMT1, divalent metal ion transporter-1; SAT1, spermidine N1-acetyltransferase 1; TfR, transferrin receptor.

The mechanism of ferroptosis is shown in Figure 1.

### 1.1. Abnormal iron metabolism

Iron is an element necessary for lipid peroxide accumulation and ferroptosis. Excessive iron load promotes the production of ROS through the Fenton reaction and iron-binding proteins, thereby promoting the occurrence of ferroptosis (4, 9, 10). Iron intake, transport, and storage affect ferroptosis (5). Under physiological conditions, intracellular iron absorption and metabolism should always be in a dynamic and stable state. Iron uptake and export proteins play important roles in the process of iron metabolism. On the one hand, transferrin receptor 1 (TFR1) and divalent metal transporter-1 (DMT1) take up extracellular iron in cells. On the other hand, ferroportin (FPN) transfers excess intracellular iron to the outside of the cell, a process that maintains the “on” and “off” state of intracellular iron homeostasis (4, 11). Iron in the daily diet is usually absorbed by intestinal epithelial cells in the form of Fe3+ and then enters cells through the transferrin receptor (TFR) on the cell membrane after binding to transferrin. Afterward, Fe3+ in cells is reduced to Fe2+ by six-transmembrane epithelial antigens of prostate 3 (STEAP3). Then, Fe2+ is released into the cytoplasmic iron pool by divalent metal transporter 1 (DMT1) or zinc–iron regulatory protein family 8/14 (ZIP8/14) to meet its own metabolic needs. Abnormal expression or dysfunction of related proteins increases the concentration of intracellular iron ions and leads to excess iron. Excess intracellular iron can generate lipid reactive oxygen species (ROS) through the Fenton and Haber–Weiss reactions, which further accumulate and initiate lipid peroxidation (LPO) to induce ferroptosis (12).

### 1.2. Abnormal lipid metabolism

Lipid metabolism is critical for regulating cellular susceptibility to ferroptosis. Because plasma membrane damage caused by iron-dependent excess accumulation is an important feature during ferroptosis (13), reducing oxidative damage from lipid peroxidation is a fundamental process to inhibit ferroptosis (14–16). ROS interacts with polyunsaturated fatty acids (PUFAs) on lipid membranes to form lipid ROS, and excessive ROS accumulation in cells induces ferroptosis (17). When PUFAs exist in large amounts, they lead to more lipid peroxides and aggravate the degree of cell ferroptosis (18). Studies found that lipoxygenases (LOXs), non-heme iron-containing proteins, are involved in the formation of iron-dependent lipid ROS. When cells contain a large amount of iron ions, they catalyze PUFAs to form lipid hydroperoxides and toxic lipid-free radicals, which cause cell damage and promote ferroptosis (19). It was later found that antioxidants significantly inhibit LOXs and further inhibit the formation of lipid hydroperoxides to prevent cell ferroptosis (19). Acyl-CoA synthetase long-chain family member 4 (ACSL4) and lysophosphatidylcholine acyltransferase 3 (LPCAT3) are involved in helping PUFAs in cell membrane synthesis and esterification to generate PUFA-PEs, which accelerate the process of ferroptosis (20, 21). Therefore, PUFA-related biosynthetic enzymes may be potential targets for regulating ferroptosis.

### 1.3. Abnormal amino acid metabolism

Ferroptosis caused by abnormal amino acid metabolism is mainly related to the abnormal metabolism of GSH. GSH is a key substance in amino acid metabolism in ferroptosis and is mainly synthesized from cysteine, glutamate, and glycine (5). Extracellular cysteine and intracellular cysteine are essential for GSH biosynthesis. Cystine generates GSH through a series of enzymatic actions, and GSH is the basic substrate for the degradation of phospholipid hydrogen peroxide (PLOOH) by glutathione peroxidase 4 (GPX4). Decreased GPX4 activity leads to the accumulation of intracellular lipid peroxides, thereby inducing ferroptosis (22, 23).

Cystine/glutamate anti-transport system Xc- (System Xc-) on the cell membrane transports extracellular cystine and intracellular glutamate in a 1:1 ratio. Cells mainly acquire cystine from the extracellular space through System Xc-, where cystine is reduced to cysteine to participate in the synthesis of GSH, and erastin acts on System Xc- to inhibit the uptake of cystine by the cell membrane, thereby reducing the synthesis of GSH and further contributing to the accumulation of ROS (24). Because glutamate is a regulator of ferroptosis and is exchanged with cystine in a 1:1 ratio by System Xc-, the concentration level of glutamate affects the function of System Xc-. A previous study reported that a high concentration of extracellular glutamate further prevents the uptake of cystine by inhibiting the biological activity of System Xc-, thereby inducing ferroptosis (11, 25). Therefore, abnormal amino acid metabolism is another important mechanism in cell ferroptosis.

### 1.4. Pathways related to ferroptosis

The process of ferroptosis is affected by different signaling pathways. As a tumor suppressor gene for cell cycle inhibition, apoptosis, and senescence, p53 is also one of the main signaling pathways of ferroptosis in cells (26, 27). Related studies showed that p53 is involved in the ferroptosis process and is a key regulator of both the canonical and non-standard ferroptosis pathways (28–30). SLC7A11 is an important part of System Xc-, and p53 acts as a transcriptional repressor of SLC7A11, participates in the process of ferroptosis, and inhibits the acquisition of cysteine by downregulating the expression of SLC7A11 and reducing GPX activity and GSH synthesis ability; this allows ROS accumulation and induces ferroptosis (28). p53 also sensitizes ferroptosis by enhancing the expression of glutaminase 2 (GLS2) and spermidine/spermine N1-acetyl-transferase 1 (SAT1) (31, 32). In addition, p53 inhibits ferroptosis by directly inhibiting the activity of dipeptidyl peptidase 4 (DPP4) or by promoting the expression of cyclin-dependent kinase inhibitor 1A (CDKN1A/p21) (33, 34).

Nuclear factor erythroid 2-related factor 2 (Nrf2) is a key regulator of cellular antioxidant activity, and its targets play important roles in iron and lipid metabolism (35, 36). Studies found that Nrf2 further inhibits ferroptosis by increasing the expression of target genes related to iron and ROS metabolism (37). Under physiological conditions, Nrf2 expression is low, and its activity is tightly regulated by Keap1 (38). When oxidative stress occurs, Nrf2 dissociates from the Keap1 cytoplasmic inhibitor and activates the Nrf2 transcriptional gene and thus plays an antioxidant role in protecting cells from oxidative stress. Activation of Nrf2 greatly reduces iron absorption and inhibits the production of reactive oxygen species, thereby enhancing cellular antioxidant capacity (15, 39–41). Also, Nrf2 stimulates the expression of GPX4, thereby inhibiting the occurrence of ferroptosis under certain circumstances (42, 43). Thus, the regulation of the Nrf2 pathway inhibits ferroptosis.

## 2. Pathogenesis of COPD

### 2.1. Pathological mechanism of COPD

The pathogenesis of COPD is based on the response of the body to inhalation of harmful particles and gases, and it is complex and has not been fully elucidated. Smoking is the main cause of COPD, and the pathogenesis of COPD is mostly related to inflammatory mediators, inherent immunity, oxidative stress, and protease/antiprotease imbalance (7, 44). Among many theories regarding the pathogenesis of COPD, ferroptosis is likely to be a major internal manifestation (8), and environmental interaction that induces respiratory inflammation is the main factor leading to the pathogenesis (45). Chronic airway inflammation and defects in epithelial repair remain core problems in COPD (46).

Cigarette smoke (CS) and harmful particles induce the body to produce highly reactive molecules such as ROS and reactive nitrogen species (RNS), thus inducing oxidative stress. The accumulation of neutrophils and macrophages in airways and pulmonary blood vessels causes the release of a large number of inflammatory mediators, which induces an inflammatory response and leads to lung tissue damage and protease/antiprotease imbalance, ultimately accelerating the progression of COPD (47). Oxidative stress causes the release of IL-1β and tumor necrosis factor-α (TNF-α) by regulating redox transcription factors such as nuclear factor kappa B (NF-κB) and activator protein 1 (AP-1), thereby enhancing the inflammatory response of lungs. It is worth noting that the increase in inflammatory cells and pro-inflammatory cytokines not only maintains the chronic inflammatory response in this population but also causes systemic damage (48).

### 2.2. Iron-dependent oxidative stress

Oxidative stress is an important pathogenic factor in COPD, and the presence of a large amount of ROS in the inflammatory response inactivates antiproteases and leads to lung tissue damage. Excessive secretion and accumulation of neutrophils lead to the production of a large amount of reactive oxygen species (ROS). The accumulation of ROS reduces the activity of histone deacetylases (HDACs) and increases the activity of histone acetyltransferases, leading to further accumulation of neutrophils, which exacerbates oxidative stress (49).

Iron homeostasis may be disrupted in inflammatory diseases, resulting in the production of excess reactive oxygen species with deleterious effects on cells and tissues. Epithelial cells and macrophages in lung tissues produce iron metabolism-related proteins, which regulate iron homeostasis and prevent the occurrence of oxidative stress (50). Disruption of pulmonary iron homeostasis is closely related to the development of COPD (51), and oxidative stress occurs due to excess iron in the lungs caused by endogenous or exogenous factors. Studies in rats showed high levels of pulmonary oxidative stress following intravenous injection of iron-containing compounds (iron dextran and iron carboxymaltose), which are mainly manifested by increased levels of nitrotyrosine and protein carbonyl modifications (52). Lung administration of Fe2O3 nanoparticles by inhalation induces ROS generation in rat lungs (53). Chio et al. found that cigarette smoke (CS) has an important effect on iron homeostasis in the lungs. Exposure of mouse and human bronchial epithelial cells to CS increases the concentrations of iron, Ft, serum ferritin, and non-heme iron in lung cells (54).

### 2.3. Lipid peroxidation

Lipid peroxidation is the loss of hydrogen atoms of intracellular lipids under the action of peroxidase or free radicals, resulting in oxidation, fragmentation, and shortening of carbon chains as well as lipid-free radicals, malondialdehyde (MDA), and 4-hydroxy-2-nonenal (4-HNE) peroxidation products, which eventually oxidatively degrade lipids and damage the lipid bilayer structure of cell membranes. (55) Currently, MDA, HNE, F2-isoprostanes (F2-isoP), and 8-iso-prostaglandin F2α (8-iso-PGF2α) are the main biomarkers for evaluating lipid peroxides.

Smoking increases the content of lipid peroxidation in the body, and lipid peroxidation is closely related to the pathological progression of COPD (56). A survey of community residents in Germany showed that the level of cotinine in plasma and the amount of smoking in community residents are proportional to the level of 8-iso-PGF2α in urine, and the survey also reported that the level of 8-iso-PGF2α in smokers is significantly higher than that in non-smokers. The study further showed that the smoking index constructed based on 71 smoking-related methylation sites has a positive dose–response relationship with the log value of 8-iso-PGF2α (57). In addition, air pollution is also a major risk factor for COPD, and air pollutants lead to elevated levels of lipid peroxides in the body. In a cohort study of 97 elderly people in the United States, Zhang et al. found that the levels of carbon monoxide, nitrogen oxides, and other related pollutants and ultrafine particulate matter (PM0.18) are closely related to the elevation of the MDA oxidative stress marker, and they also reported that the effect of the component with a smaller particle size is stronger. (58) A survey of adults commuting on the highway for 3 h during the morning rush hour in Atlanta reported that exhaled nitric oxide, C-reactive protein, and MDA levels of patients with asthma and without asthma are significantly higher than baseline levels, indicating that the inhalation of harmful gases causes pulmonary inflammation and oxidative stress (59).

## 3. Role of ferroptosis in COPD

**Figure 2:**
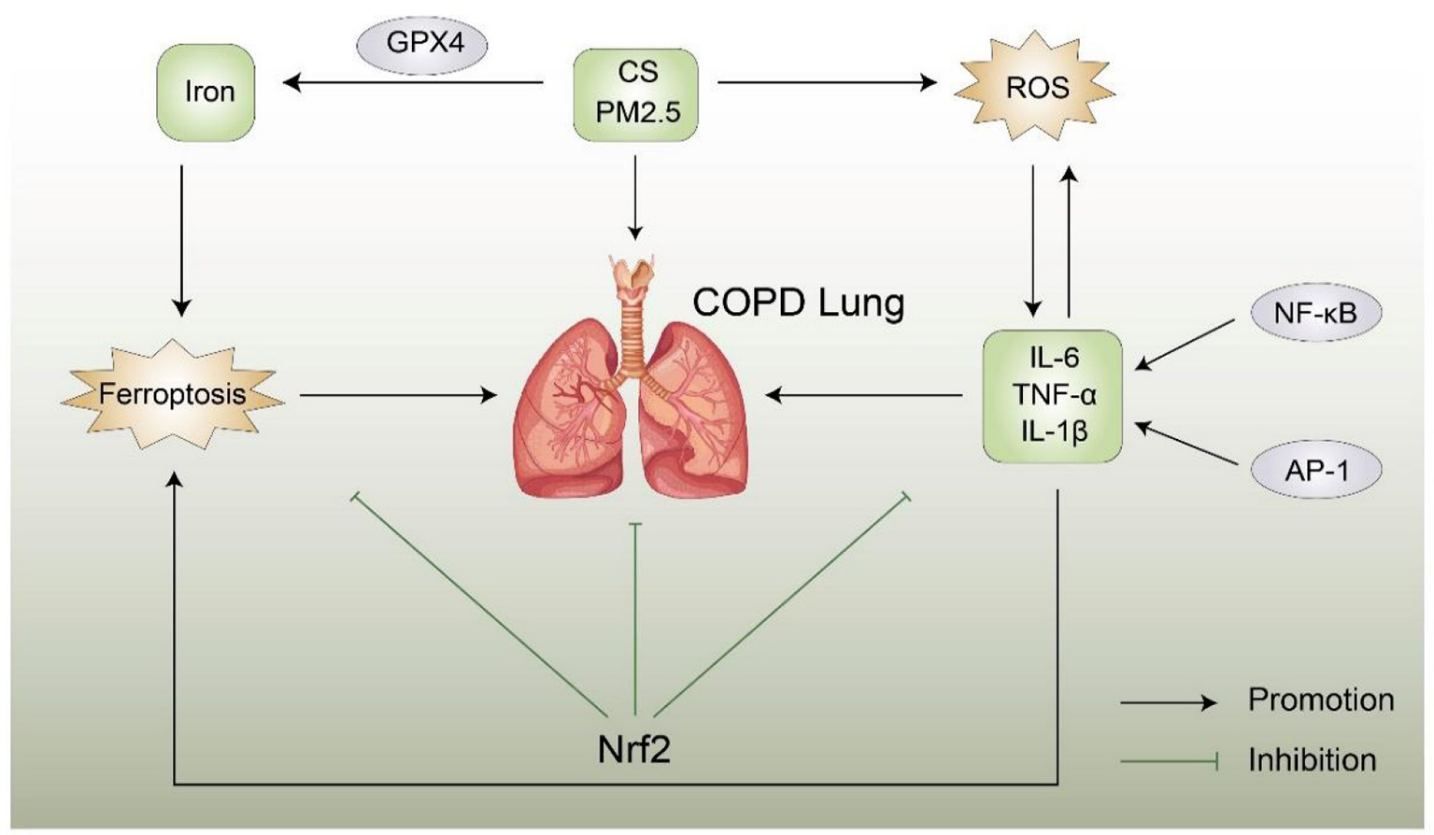
Possible relationship between ferroptosis and COPD, ROS, CS, and PM2.5 trigger COPD, resulting in an inflammatory reaction and abnormal levels of inflammatory factors. Conversely, abnormal inflammatory factors can aggravate COPD, Nrf2 can inhibit the production of reactive oxygen species and iron and the occurrence of COPD and ferroptosis. A direct link between ferroptosis and COPD remains unclear. Nrf2, Nuclear factor erythroid 2-related factor 2; CS, cigarette smoke; ROS, reactive oxygen species; AP-1, activator protein 1; NF-κB, nuclear factor of kappa B.

The role of ferroptosis in COPD is shown in Figure 2.

Ferroptosis is involved in the pathogenesis of COPD in a COPD mouse model. Murine lung epithelial cells exposed to CS exhibit unstable iron accumulation and increased lipid peroxidation accompanied by a non-apoptotic mode of cell death negatively regulated by GPX4. In addition, the mouse model further confirmed that the treatment of lung epithelial cells with deferoxamine and Fer-1 effectively reduces the lipid peroxidation induced by CSE, and inhibition of GPX4 also has the same effect (8). Therefore, related inhibitors such as deferoxamine and Fer-1 are potential approaches for the prevention of ferroptosis and COPD treatment. PM2.5 is one of the pathogenic factors of COPD. Studies found that, after inhalation of PM2.5 particles, the iron content and ROS concentration in human endothelial cells significantly increased, whereas the expression of GSH and NADPH decreased; in addition, the changes in the expression of TfR and Ft lead to an imbalance in cellular iron homeostasis, thereby inducing ferroptosis. The use of Fer-1 and deferoxamine improves GSH and nicotinamide adenine dinucleotide phosphate (NADPH) levels (60). Tang et al. found that cigarette smoke extract (CSE) aggravates the damage and death of BEAS-2B cells as well as increases the levels of IL-6 and TNF-α inflammatory factors, resulting in iron property changes. *In vivo* studies showed that CS causes lung injury in COPD rats, increases inflammatory cell infiltration, increases inflammatory cytokine secretion, and induces ferroptosis in lung tissue cells of COPD rats. *In vitro* and *in vivo* studies showed that CSE/CS increases the MDA content, increases the iron content, downregulates GPX4, decreases ferritin heavy chain levels, and upregulates transferrin receptor levels and also reported that curcumin reverses CS-induced lung inflammatory damage and epithelial cell ferroptosis (61).

Morphological aspects of ferroptosis mainly manifest as mitochondrial shrinkage, reduction or disappearance of mitochondrial cristae, and increased mitochondrial membrane density. Liu et al. found that dihydroquercetin (DHQ) inhibits CS-induced ferroptosis in the pathogenesis of COPD by activating the Nrf2-mediated pathway and attenuating CSE-induced mitochondrial morphological changes (62). *In vitro* and *in vivo* studies reported that the mRNA and protein expression levels of SLC7A11 and GPX4 are increased after DHQ treatment, and they also demonstrated that CSE-induced lipid peroxidation in HBE cells is significantly reduced after DHQ treatment. In addition, DHQ reverses CSE-induced excess MDA and ROS production, and it has also been reported that the DHQ-induced increase in SLC7A11 and GPX4 mRNA and protein levels is reversed by the use of an Nrf2-specific inhibitor (ML38.5). These findings suggest new options for the treatment of patients with COPD.

Nrf2 is a key factor in maintaining the oxidative/antioxidative balance, which can prevent the occurrence of COPD by resisting oxidative stress and lung inflammation. Recent studies reported that CpG hypermethylation in the promoter causes downregulation of Nrf2 expression in the lung tissue of patients with COPD (65). Zhang et al. found that, in HBE cells treated with CSE, the expression levels of reactive oxygen species (ROS), lipid peroxides, and MDA are increased, and they also reported that the levels of IL-1β and IL-8 are increased but that the expression levels of GPX4 and SOD are decreased, indicating that CSE induces ferroptosis in HBE cells and increases the release of inflammatory factors (63). However, increasing the expression of Nrf2 enhances the expression of GPX4 and SOD but inhibits the expression of ferroptosis and related inflammatory factors in the supernatant. Further studies found that CS-/CSE-induced hypermethylation may lead to abnormal expression of Nrf2, and targeting methylation and ferroptosis may prevent the progression of COPD, which may be a new strategy for the treatment of COPD in the future.

Macrophages are innate immune cells that play a key role in alleviating inflammation and defending against invasion by external pathogens (66, 67). Studies found that macrophage activation is closely related to the occurrence of COPD, and the total number of macrophages is proportional to the severity of smoking and COPD (68). Using both *in vitro* and *in vivo* studies, Liu et al. found increased levels of M2 macrophages, MMP9 expression, and MMP12 expression in patients with COPD, CS-exposed mice, and THP-M cells cocultured with CSE-treated human bronchial epithelial (HBE) cells, suggesting that NCOA4 and ferroptosis are involved in the pathogenesis of COPD (64). This trend is further reversed using NCOA4 siRNA and the ferrostatin-1 ferroptosis inhibitor. Therefore, blocking NCOA4 may be a promising therapeutic strategy for COPD, which provides a new direction for COPD diagnosis and treatment research. The Mechanism of ferroptosis in COPD is shown in Table 1.

**Table 1:** Mechanism of ferroptosis in COPD.

**Reagents**	**Model**	**Key mechanisms**	**References**
DFO, Fer-1	HBEC; COPD mouse model	CS promotes the accumulation of unstable iron *via* NCOA4-mediated ferritinophagy, inducing lipid peroxidation and ferroptosis	(8)
Fer-1, DFOM	EA.hy 926; HUVECs	PM2.5 causes iron overload and oxidative stress and further induces ferroptosis.	(60)
Fer-1, DFO, CUR	BEAS-2B; COPD mouse model	CS/CSE causes cell damage and enhances inflammation, and oxidative stress induces ferroptosis	(61)
Dihydroquercetin (DHQ); ML385,	HBE cells; COPD mouse model	CSE induces cellular oxidative stress and ferroptosis	(62)
Fer-1	HBE cells; COPD mouse model	CSE induces increased levels of cellular ROS, lipid peroxides, and MDA, with IL-1β and IL-8 inducing ferroptosis and inflammatory responses, respectively	(63)
Fer-1	HBE/THP-M cells; COPD mouse model	NCOA4-induced ferroptosis promotes macrophage M2 polarization	(64)

## 4. Ferroptosis as a potential therapeutic strategy in COPD

The imbalance of iron absorption and metabolism is an important factor affecting the progression of COPD. Therefore, correcting the local metabolism of iron is another key method for the treatment of COPD. Studies showed that iron chelators, antioxidants, iron supplementation, and dietary restriction are effective ways to treat COPD.

### 4.1. Iron chelators

Common iron chelators include deferoxamine (DFO), deferiprone, and deferasirox. DFO is one of the drugs that have been approved by the FDA for the treatment of iron overdose (69). In addition, a previous study found that DFO reduces the levels of inflammatory factors and reactive oxygen species *in vitro* and that it exerts an anti-inflammatory effect and reduces the inflammatory response (70). However, genetic factors are one of the important factors in the complex pathogenesis of COPD (71). Another study showed that iron regulatory protein 2 (IRP2, also known as IREB2) is increased in the lung tissue of patients with COPD, and IRP2 has been identified as a COPD susceptibility gene (72). Through COPD model mouse experiments, Cloonan et al. found that mice lacking the IRP2 gene are protected from CS-induced COPD, and they identified IRP2 as a regulator of mouse lung mitochondrial function. They further found that mice treated with a mitochondrial iron chelator (deferiprone) or fed a low-iron diet are protected from CS-induced COPD; in addition, CS-induced mucociliary clearance (MCC) impairment, lung inflammation, and lung injury in mice are attenuated (73). Therefore, these findings suggest that mitochondrial iron chelators may be a new potential approach for treating COPD.

### 4.2. Antioxidants

Oxidative stress is an important cause of airway inflammation, lung parenchyma destruction, and lung function decline in COPD, mainly induced by inhalation of air pollutants such as CS/CSE and dust. The acute exacerbation of COPD is closely related to oxidative stress and oxidant/antioxidant imbalance in the blood (74). Studies showed that H2S-activated Nrf2 pathway-mediated antioxidant effects play an important role in the progression of COPD (75). Wang et al. found that, after PM2.5 treatment of airway epithelial cells, the mitochondrial membrane density increases and characteristic changes in ferroptosis occur. Furthermore, COX2 expression is increased in patients with COPD. In the PM2.5-mediated mouse model and cell injury model, the levels of LIP ROS, total ROS, and MDA increased, and the levels of GSH, GSH Px, and GPX4 antioxidants decreased, indicating that PM2.5-mediated ferroptosis is involved in the pathogenesis of COPD (76). Further studies found that H2S inhibits lipid peroxidation-mediated ferroptosis by restoring the redox balance and regulating the Nrf2–PPAR ferritin in the phagocytosis pathway, thereby reducing PM2.5-induced emphysema and airway inflammation. These results suggest that inhibition of ferroptosis may be a potential therapeutic target for diseases with oxidative stress as the core pathogenesis, and H2S may be a potential antioxidant that blocks PM2.5 and causes COPD pathogenesis.

## 5. Conclusion and outlook

Ferroptosis, a new type of cell death, is involved in the pathogenesis of various diseases. Ferroptosis is a process involving abnormal metabolism of iron, amino acids, and lipids, which are involved in cell proliferation and differentiation. The metabolic process of ferroptosis is complex, and its mechanism has been preliminarily studied. Both *in vitro* and *in vivo* studies showed that ferroptosis is closely related to a variety of disease processes, and appropriate intervention in ferroptosis can treat the disease and delay the progression of related diseases. Although some progress has been made in the role of ferroptosis in lung cancer, ALI, and pulmonary fibrosis, the role of ferroptosis in COPD is not fully understood, and further clinical and experimental studies are needed. Although the existing ferroptosis inducers are effective, the therapeutic modalities for ferroptosis are still insufficient and only include iron inhibitors, iron chelators, and antioxidants. Identifying effective diagnostic markers of COPD may aid in the treatment of ferroptosis in COPD. For example, the known important regulator of iron metabolism, hepcidin, and its receptor, Fpn1, have been demonstrated to be effective diagnostic markers for COPD, and they may be promising targets for future drug development. In conclusion, with increasing studies on ferroptosis, new related regulators and their functions are constantly being explored. Considering ferroptosis as an entry point to develop COPD treatment regimens and targeted drugs has important clinical value.

## Data availability statement

The original contributions presented in the study are included in the article/supplementary material, further inquiries can be directed to the corresponding authors.

## Author contributions

DM and CZ wrote the manuscript. RJ and ZL provided language assistance. WW proofread the manuscript. SS was involved in the design and review of this study. All authors contributed to the article and approved the submitted version.
